# The nerve conduction variables in relation to academic performance among male medical students at King Saud bin Abdulaziz University for Health Sciences: an exploratory observational study

**DOI:** 10.3389/fnhum.2026.1855183

**Published:** 2026-06-11

**Authors:** Awad M. Almuklass, Omar M. Al Mutlaq, Muhannad Alharbi, Ibrahim Alojaymi, Abdullah AlSharif, Qusai Alhazmi

**Affiliations:** 1 Department of Basic Medical Sciences, College of Medicine, King Saud bin Abdulaziz University for Health Sciences (KSAU-HS), Riyadh, Saudi Arabia; 2 King Abdullah International Medical Research Center (KAIMRC), Riyadh, Saudi Arabia; 3 Ministry of National Guard - Health Affairs, Riyadh, Saudi Arabia

**Keywords:** academic indicators, medical students, nerve conduction latency, nerve conduction velocity, reaction time

## Abstract

**Introduction:**

Peripheral nerve conduction parameters vary between individuals and may reflect differences in peripheral neurophysiological function. This exploratory observational study examined whether ulnar nerve conduction measures and reaction time were associated with academic indicators among male medical students. The study was designed to provide preliminary evidence regarding possible associations between peripheral electrophysiological measures and academic performance among medical students.

**Methods:**

A total of 54 male medical students at King Saud bin Abdulaziz University for Health Sciences (mean age: 21.26 ± 0.76 years) were enrolled in this observational study. Ulnar nerve function was evaluated using standard nerve conduction tests with a focus on latency and velocity through the wrist and elbow. Reaction time was assessed using a computerized task. Academic measures including university GPA, high school GPA, the General Aptitude Test (GAT), and the Scholastic Achievement Admission Test (SAAT) scores, were recorded. Associations were assessed using Spearman’s or Pearson’s correlation, as appropriate based on normality testing (Shapiro–Wilk).

**Results:**

The primary analysis did not support an association between ulnar nerve velocity and university GPA. All statistically significant findings were from secondary exploratory analyses and were not corrected for multiple comparisons. Statistical analyses indicated an exploratory negative correlation between wrist latency and high school GPA (*r* = −0.319, *p* = 0.018) and university GPA showed a preliminary negative correlation with age (*r* = −0.291, *p* = 0.032). A significant positive correlation between university GPA and SAAT scores was observed (*r* = 0.272, *p* = 0.046). Moreover, SAAT score showed an exploratory positive association with elbow latency (*r* = 0.329, *p* = 0.015). The mean reaction time was 254.0 ms and showed no statistically significant correlations with GPA, GAT, or SAAT scores. In addition, academic indicators were self-reported which may introduce recall and social desirability bias. Nevertheless, the findings suggest that some academic indicators, such as high school GPA, are possibly correlated with neural conduction traits.

**Discussion:**

These initial findings suggest exploratory associations between selected peripheral nerve conduction measures and academic indicators. Importantly, all secondary significant findings were uncorrected for multiple comparisons and should be treated as preliminary. Future research in additional subjects and verified records is justified to explore this relationship further.

## Introduction

The rapid and accurate transmission of nerve impulses is fundamental to essential cognitive processes such as memory, attention, and reasoning. Although peripheral nerve conduction does not directly measure central processing, it provides a standardized electrophysiological index of axonal conduction properties that may vary between individuals. Nerve conduction velocity (NCV), largely determined by axonal myelination, ensures efficient communication between brain regions and may contribute to efficient neural transmission and has been discussed in relation to cognitive and functional performance (Waxman, 1980; Fields, 2008). Neuroanatomical features also influence information processing; neurons with larger dendritic arbors integrate richer synaptic input, and variations in dendritic complexity and action potential speed have been directly linked to differences in intelligence (Jacobs et al., 1993; DeFelipe, 2011; Chklovskii et al., 2004; Goriounova et al., 2018). Faster nerve conduction is associated with superior cognitive performance, whereas slower peripheral conduction has been observed in individuals with Alzheimer’s disease (Qian et al., 2022). In addition, differences in cognitive reserve have been shown to modulate brain responses during demanding memory tasks (Scarmeas et al., 2003). This raises the question of whether peripheral electrophysiological measures may show measurable associations with academic indicators.

Early investigations demonstrated a positive correlation between brain nerve conduction velocity and intelligence in neurologically normal adults (Jensen, 1992). Later work reported that faster median nerve conduction velocities were linked to higher intelligence scores (Vernon and Mori, 1992). More recent findings from twin studies suggested that the modest correlation between NCV and cognitive ability is largely explained by shared genetic influences (Rijsdijk and Boomsma, 1997). Therefore, this evidence positions NCV as a peripheral measure that may be associated with aspects of cognitive or functional performance, reflecting both inherited potential and vulnerability to decline.

Given these findings, it remains unclear whether such physiological markers align with academic achievement in real-world settings. In Saudi Arabia, admission to competitive programs such as medicine depends substantially on high school GPA and national standardized tests, including the Scholastic Achievement Admission Test (SAAT; *Tahsili*) and the General Aptitude Test (GAT; *Qudrat*). Although admissions decisions rely strongly on these measures, the predictive validity of these assessments is inconsistent. Some studies report moderate associations with preclinical GPA (Althewini and Al, 2022), while others show that their combined contribution, even when English proficiency is included, explains only a modest proportion of the variance in student outcomes (Althewini, 2019; Alkushi and Althewini, 2020). Additional research also indicates that although GAT and SAAT are statistically significant, their predictive power remains limited (Althewini, 2019; Alkushi and Althewini, 2020). The overall evidence suggests that while these tests remain important for selection, they are insufficient as predictors of long-term academic success, highlighting the need for complementary measures. Therefore, physiological measures may offer an objective complement less influenced by curriculum and grading variability.

Importantly, both standardized admission tests and college GPA have been widely regarded as proxies for general cognitive ability, reflecting aspects of fluid and crystallized intelligence (Deary et al., 2007; Rohde and Thompson, 2007). Prior research has shown that measures such as aptitude tests, achievement scores, and academic performance correlate strongly with intelligence test results and are often used as indirect indicators of intellectual capacity in educational and occupational settings (Deary et al., 2007; Rohde and Thompson, 2007).

Academic achievement is also influenced by a variety of psychosocial and physiological factors, including emotional regulation, stress, and sleep quality (MacCann et al., 2019; Perera and DiGiacomo, 2013; Maslach and Leiter, 2008; Curcio et al., 2006). Among Saudi medical students, stress has been shown to be strongly associated with poor sleep quality, further highlighting how these factors interact to impair academic performance (Almojali et al., 2017). These domains have been extensively studied, yet whether direct physiological measures of neural efficiency can also serve as predictors of academic performance remains largely unexplored. Most previous investigations have relied on psychometric testing or neuroimaging to assess brain efficiency, but electrophysiological markers of peripheral nerve transmission have rarely been considered in educational contexts (Waxman, 1980; DeFelipe, 2011). Considering the role of neurophysiology in nerve conduction velocity and the lack of prior research on students’ academic performance in Saudi Arabia, the hypothesis was that nerve conduction measurements, especially velocity, would be associated with academic performance. This study aimed to examine whether NCV may add value to existing academic and psychosocial measures by assessing its association with academic performance in medical students.

## Methods

### Study design and setting

This was an exploratory, observational, correlational study designed to examine the associations between electrophysiological measures of peripheral nerve conduction and academic performance among medical students. The observational approach was chosen because it allows the collection of multiple physiological and academic variables at a single point in time, making it efficient and suitable for identifying associations without the need for longitudinal follow-up.

The study was conducted in the Physiology Laboratory, College of Medicine, King Saud bin Abdulaziz University for Health Sciences (KSAU-HS), Riyadh, Saudi Arabia. The College, established in 2005 within King Abdulaziz Medical City, provides access to standardized electrophysiology equipment, controlled laboratory conditions, and a medically homogeneous student population.

### Participants

#### Eligibility criteria

The target population consisted of male medical students enrolled at KSAU-HS aged 18–25 years.

- Inclusion criteria: Healthy male students within the target age range, who were able to complete laboratory tasks and provide academic records.
- Exclusion criteria: Students with a BMI > 30 (corresponds to obesity), a history of neurological disorders or seizures, motor impairments, previous upper-limb fractures or surgeries, or documented low serum vitamin B12 levels. These exclusions were applied to minimize confounding by medical conditions that could alter nerve conduction or dexterity.

#### Recruitment and exclusions

From an estimated pool of ~160 eligible students, 69 volunteered to participate. Screening was performed using a medical history questionnaire, BMI measurement, and a review of prior medical records when available. After applying exclusion criteria, 15 students (21.7%) were excluded:

- Two due to obesity (BMI > 30),
- Two due to neurological or motor impairments,
- Three due to data entry technical issues, (the file closed after data gathering without autosave and due to ethical consideration, they were excluded without recruiting them again) and it was pre analysis.
- One due to age,
- Seven due to missing academic data.

The final analyzable sample was *n* = 54 (see Table 1).

**Table 1:** Participant selection summary (STROBE-aligned flow).

Estimated eligible male medical students, *n* ≈ 160
Volunteered / assessed for eligibility (*n* = 69)
Excluded before analysis (*n* = 15)
Reasons for exclusion: Obesity, BMI > 30 kg/m^2^ (*n* = 2) Neurological or motor impairments (*n* = 2) Technical data-entry problems (*n* = 3) Age outside eligibility range (*n* = 1) Missing academic data (*n* = 7)
Included in final analysis (*n* = 54)

### Sample size and power

*A priori* power analysis was conducted using *GPower (version 3.1)*. Assuming a correlation coefficient of *r* = 0.40, *α* = 0.05, three predictors, and 80% power, the required minimum sample size was *n* = 66. Although the final sample (*n* = 54) was below the target sample size, the study retained adequate power to detect moderate correlations (*r* ≥ 0.36–0.38), while being underpowered for smaller associations. To maintain inferential clarity, the relationship between ulnar nerve conduction velocity at rest and university GPA was pre-specified as the primary analysis. All other associations were treated as exploratory and interpreted with caution.

### Data collection procedures

All procedures were conducted in a standardized sequence during a single laboratory visit. Prior to the main study, a pilot test involving eight participants was performed to refine electrode placement, test instrument calibration, and ensure repeatability.

1. Screening and consent

Eligibility screening was completed first. Participants were informed verbally and in writing about the study’s purpose, procedures, and potential risks (e.g., mild discomfort during nerve stimulation). Written informed consent was obtained before participation. Each participant was assigned a coded serial number, which was used throughout data entry and analysis to preserve anonymity. Academic records were handled by an investigator who had no role in laboratory testing to avoid bias in linking academic and physiological data.

1. Demographics and handedness

Demographic information included age, height, and weight. BMI was calculated (kg/m^2^). Hand dominance was assessed using the Edinburgh Handedness Inventory (EHI), a validated 10-item scale (Veale, 2014). Each participant’s laterality quotient was calculated to confirm hand-dominance classification. Dominant-hand status was essential because the Grooved Pegboard Test (GPT) was performed with the dominant hand, whereas the ulnar nerve conduction study (NCS) was standardized to the non-dominant hand.

1. Nerve conduction study (NCS)

Nerve conduction studies of the ulnar nerve were performed using a PowerLab acquisition system (ADInstruments, Australia). Participants were seated upright with a neutral spine posture, hips flexed at 90–110°, and the non-dominant arm supported on a padded rest to minimize movement artifacts. The skin was cleaned with alcohol wipes to reduce impedance.

**Figure 1:**
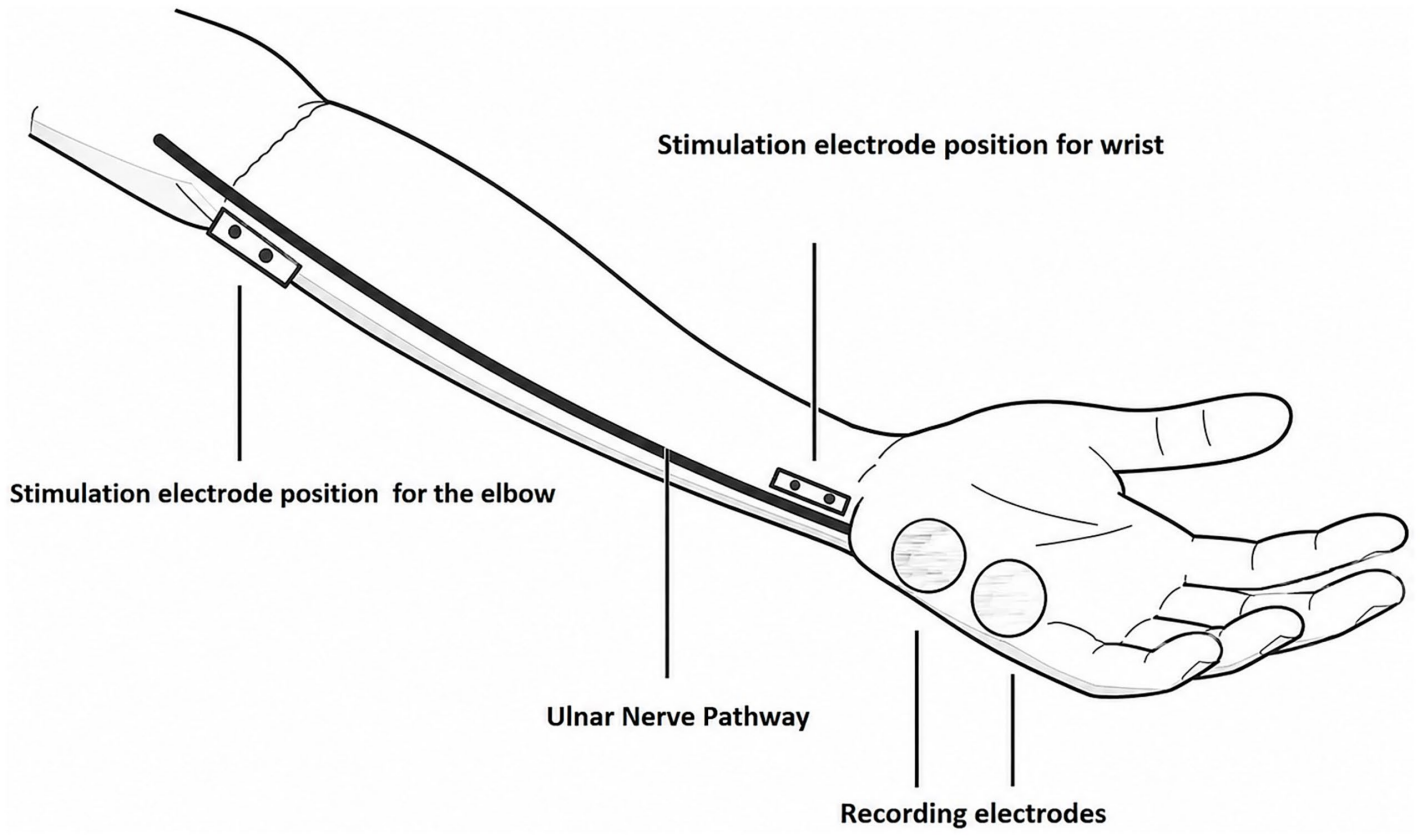
Schematic illustration of the ulnar nerve conduction study setup. The pathway of the ulnar nerve is shown with stimulation electrodes positioned at the medial wrist and ulnar groove at the elbow.

- Electrode placement: The cathode and anode electrodes were positioned over the hypothenar muscles on the medial palm just below the little finger. The ground electrode was placed on the middle anterior forearm, just below the cubital fossa (Almuklass et al., 2024). Conductive electrode cream was applied at each site (Figure 1).
- Stimulation sites: (1) the medial wrist and (2) the ulnar groove under the elbow. Each site was marked, and the inter-electrode distance was measured precisely using a rigid ruler.
- Stimulation intensity: 20 mA, adjusted to achieve supramaximal stimulation.
- Recordings: Six trials were performed for each participant: two at rest (baseline), two during the GPT task (dominant hand performing), and two immediately after task completion.
- Outcomes recorded: Conduction velocity, wrist latency, and elbow latency were automatically calculated by the PowerLab software.

The ulnar nerve conduction study (NCS) was standardized to the non-dominant hand, while the motor task was performed with the dominant hand. This setup was chosen to avoid movement artifacts that would compromise signal quality during the high-dexterity task. Furthermore, this design allowed for the assessment of NCV as a proxy for systemic neural efficiency. This approach is supported by previous findings in similar cohorts, showing no significant intra-individual differences in ulnar nerve conduction parameters between the dominant and non-dominant hands (Almuklass et al., 2024).

1. Grooved Pegboard Test (GPT)

**Figure 2:**
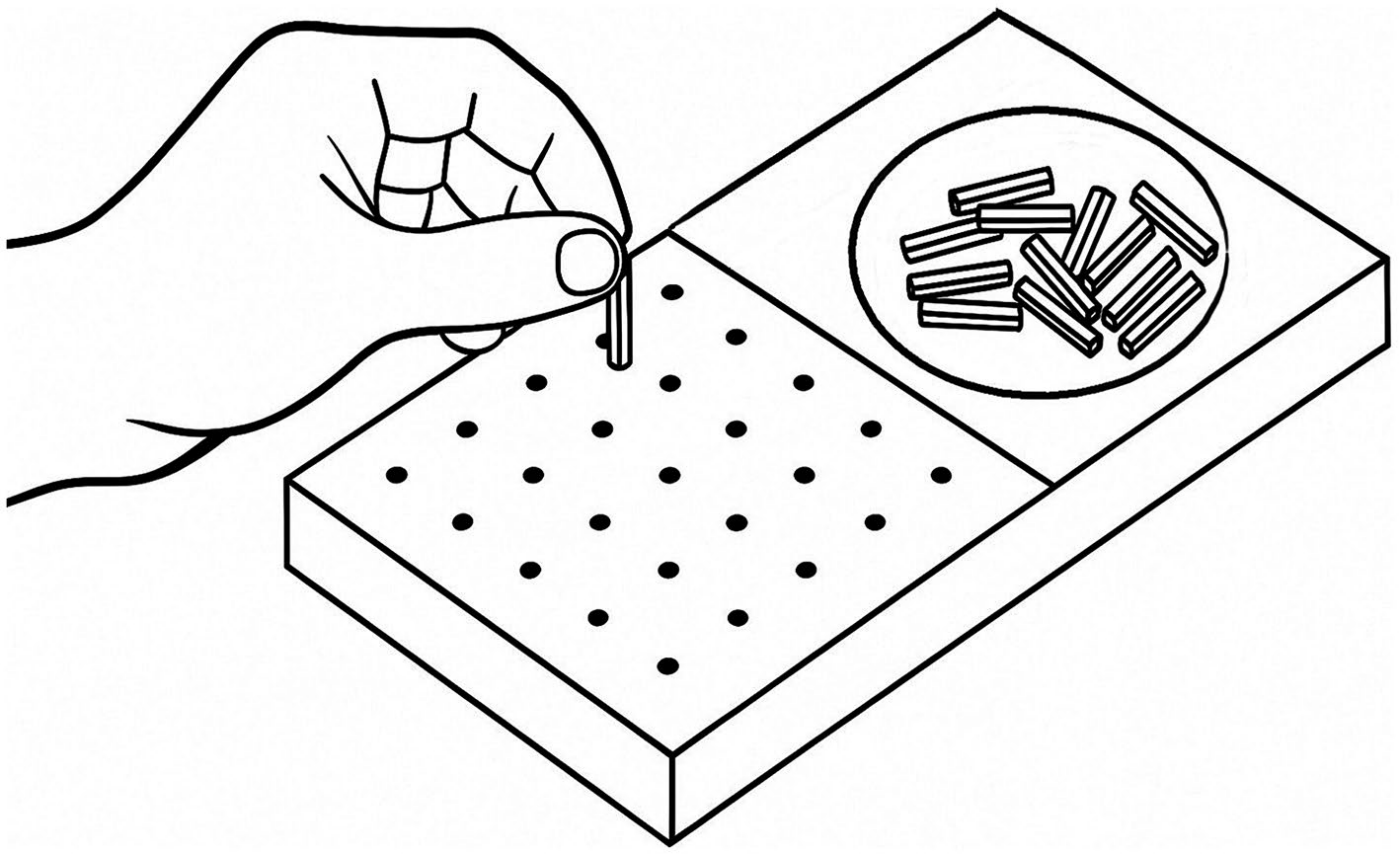
Schematic illustration of the Grooved Pegboard Test (GPT). The board contains 25 slotted holes arranged in a 5 × 5 matrix, with a circular container holding key-shaped pegs.

The GPT was performed using the Lafayette Instrument Grooved Pegboard. Participants, using their dominant hand, inserted 25 slotted pegs into the corresponding holes as quickly as possible, rotating each peg as necessary. Task instructions were standardized and demonstrated once by the investigator. Timing began with the insertion of the first peg and ended with the final peg, measured using a calibrated stopwatch (precision ±0.01 s). During the GPT, concurrent NCS recordings were collected from the non-dominant hand, linking motor-task performance with peripheral nerve conduction (Figure 2) (Almuklass et al., 2024; Almuklass et al., 2016).

1. Reaction time task

Reaction-time tasks are widely used as sensitive indices of information-processing speed and performance (Sauerland et al., 2024). Simple visual reaction time was measured using Inquisit 6 software (Millisecond Software, USA) on a laptop computer (Millisecond Software, 2024). Participants were seated upright in a standardized posture (spine neutral, hips at 90–110°, knees and ankles at right angles, feet flat on the floor). On each trial, a black fixation cross appeared on the screen for a variable interval (1–3 s) before abruptly changing to a red circle. Participants were instructed to press the spacebar as quickly as possible upon stimulus change. Each participant completed 20 trials, and the mean was calculated. To enhance reliability, the full test was repeated twice, with a 1–2 min rest between runs; the average of both repetitions was used for analysis.

1. Academic performance data

Academic indicators were obtained via a self-administered questionnaire. Students reported:

- High school GPA (percentage),
- University GPA (on a 5-point scale),
- Scholastic Achievement Admission Test (SAAT; *Tahsili*) score,
- General Aptitude Test (GAT; *Qudrat*) score.

To protect privacy and reduce bias, students were instructed that they could omit responses or indicate “do not know.” Completed forms were sealed in envelopes labeled only with the participant’s serial number and submitted directly to the academic-data investigator. To minimize order effects, participants were randomly assigned to one of two groups: half (*n* = 27) completed the academic forms before physiological testing, and the other half afterward.

### Data handling and confidentiality

All data were entered into a coded database using participant serial numbers only. The investigator responsible for academic data was not involved in laboratory testing or direct contact with participants. Data were stored securely on password-protected computers, accessible only to the study team.

### Statistical analysis

Data analysis was conducted with IBM SPSS Statistics version 24. Continuous variables were expressed as mean ± standard deviation. The Shapiro–Wilk test was used to assess normality.

Because this was an exploratory study, no correction for multiple comparisons was made. Therefore, statistically significant secondary findings should be interpreted cautiously.

- Associations: Spearman’s correlation was used for non-normal distributions and Pearson’s correlation for normal distributions.
- Effect sizes: correlations of |r| ≥ 0.40 were interpreted as moderate.
- Significance: two-tailed *p* < 0.05 was considered statistically significant.
- Precision: 95% confidence intervals were calculated for all effect estimates.
- Primary analysis: correlation between ulnar nerve conduction velocity at rest and university GPA.
- Secondary analyses: associations of latency, GPT completion time, and reaction time with academic indicators.

### Ethical considerations

This study was reviewed and approved by the Institutional Review Board (IRB) of King Abdullah International Medical Research Center (KAIMRC) (Approval No. 0000040224). All participants were adult students who provided written informed consent prior to enrollment. The study was carried out in accordance with the ethical principles of the Declaration of Helsinki (2013 revision). Participation was voluntary, and the confidentiality of all personal and academic data was strictly maintained throughout the study.

## Results

Out of 69 male participants invited to take part in the study, 54 were recruited, giving a response rate of 78.3%. The Shapiro–Wilk test was conducted to assess the distributional assumptions of the core demographic and academic variables across the sample. The analysis revealed that Age (*W* = 0.727), BMI (*W* = 0.946), University GPA (*W* = 0.808), High School GPA (*W* = 0.611), and SAAT scores (*W* = 0.928) all demonstrated significant departures from a normal distribution (*p* < 0.05). Conversely, GAT scores maintained a normal distribution profile (*W* = 0.958, *p* = 0.054). This widespread violation of normality parameters mathematically justifies the decision to prioritize non-parametric statistical methods, specifically Spearman’s rank correlation for evaluating the primary relationships involving these skewed variables throughout the study.

All respondents were high school graduates aged between 20 and 23 years. The majority had a normal (*n* = 22, 40.7%) or overweight (*n* = 24, 44.4%) body mass index. Most participants were right-handed (*n* = 48, 88.9%) and reported no history of chronic medical conditions. In terms of height, 33 participants (61%) measured between 170 and 179 cm. Arm length measurements showed that 34 participants (63%) fell within the 220–240 mm category. These results provide a clear overview of the demographic and physical characteristics of the study sample (Table 2).

**Table 2:** Descriptive and physical characteristics.

Category	Subcategory	Frequency (*n*)	Percentage (%)
Age	20–23 years	54	100
Chronic disease	No	54	100
BMI	Underweight	8	14.8
	Normal	22	40.7
	Overweight	24	44.4
	Obese	0	0
Handedness	Right	48	88.9
	Left	5	9.3
Height	<160 cm	1	1.9
	160–169 cm	12	22.2
	170–179 cm	33	61.1
	180–189 cm	8	14.8
	190 + cm	0	0

BMI, body mass index; cm, Centimeter; mm, Millimeter.

A total of 15 participants (21.7%) were excluded from the final analysis as they did not meet the inclusion criteria. Reasons for exclusion included a history of chronic illness, prior surgeries, neurological or motor impairments, or obesity (BMI > 30). To ensure the consistency and reliability of the study outcomes, only participants who met the full eligibility criteria were included in the final dataset.

Descriptive statistics were computed for the main variables under investigation, including nerve conduction velocities across three time points (before, during, and after Pegboard test), academic performance indicators (University GPA and High School GPA), and standardized test scores (GAT and SAAT). The mean ulnar nerve conduction velocity was 54.54 ± 5.79 m/s before, 54.66 ± 5.65 m/s during, and 53.95 ± 5.73 m/s after the Pegboard test, indicating minor but consistent fluctuations over the testing phases (Table 3).

**Table 3:** Descriptive and academic characteristics.

Statistic	Velocity before	Velocity during	Velocity after	Uni_GPA	HS_GPA	GAT	SAAT
Mean	54.54	54.66	53.95	4.34	98.92	92.66	90.15
SD	5.79	5.65	5.73	0.47	1.58	3.59	4.63

SD, standard deviation; Uni_GPA, university grade point average; HS_GPA, high school grade point average; GAT, General Aptitude Test (Qudrat); SAAT, Scholastic Achievement Admission Test (Tahsili).

In regard to the academic performance metrics, the mean University GPA was 4.35 ± 0.48 (on a 5.0 scale), reflecting a moderate degree of performance variability among participants. High school GPA showed less dispersion, with a mean of 98.92 ± 1.58 (on a 100-point scale), indicating a generally high-performing cohort. Standardized test results showed a mean GAT score of 92.66 ± 3.59 and a mean SAAT score of 90.15 ± 4.63. These scores support the characterization of the study sample as academically high-performing (Table 3).

In this study, we investigated the relationship between academic performance and nerve conduction studies of the ulnar nerve. However, the prespecified primary analysis showed no statistically significant association between ulnar nerve velocity and university GPA. Therefore, secondary associations were considered exploratory. Spearman’s correlation analysis revealed several statistically significant associations. High school GPA was negatively correlated with wrist latency both during the Pegboard test (*r* = −0.319, 95% CI [−0.541, −0.056], *p* = 0.018) and after the test (*r* = −0.291, 95% CI [−0.518, −0.025], *p* = 0.032) (Table 4). The SAAT score demonstrated a positive correlation with both University GPA (*r* = 0.272, 95% CI [0.005, 0.503], *p* = 0.046) and elbow latency, specifically before (*r* = 0.329, 95% CI [0.067, 0.549], *p* = 0.015) and after (*r* = 0.305, 95% CI [0.041, 0.530], *p* = 0.025) the Pegboard task (Table 5). University GPA also showed a negative correlation with age (*r* = −0.291, 95% CI [−0.518, −0.025], *p* = 0.032) and weak to no correlation with nerve function. Reaction time was not statistically significantly correlated with University GPA (*r* = −0.085, *p* = 0.538), SAAT (*r* = −0.080, *p* = 0.562), and GAT (*r* = −0.150, *p* = 0.278) (Table 6). The GAT score did not display any statistically significant correlations with nerve conduction or academic performance metrics.

**Table 4:** HS GPA correlation with nerve function.

Nerve conduction variable	Correlation coefficient	95% CI	*p*-value
b BEL (s)	−0.173	[−0.421, 0.099]	0.208
b BWL (s)	−0.117	[−0.373, 0.156]	0.398
b BL (s)	−0.033	[−0.298, 0.237]	0.811
b BV (m/s)	0.197	[−0.075, 0.441]	0.152
d BEL (s)	−0.222	[−0.462, 0.049]	0.105
d BWL (s)*	−0.319*	[−0.541, −0.056]	0.018
d BL (s)	0.125	[−0.148, 0.380]	0.364
d BV (m/s)	0.052	[−0.219, 0.316]	0.705
a BEL (s)	−0.241	[−0.478, 0.028]	0.078
a BWL (s)*	−0.291*	[−0.518, −0.025]	0.032
a BL (s)	0.094	[−0.178, 0.353]	0.497
a BV (m/s)	0.092	[−0.180, 0.351]	0.507

HS_GPA, high school grade point average; B, best reading; b, before pegboard test; d, during pegboard test; a, after pegboard test; (m/s), meter per second; (s), second; E, elbow; W, wrist; V, velocity; L, latency.

*Statistically significant (*p* < 0.05) correlation coefficients.

**Table 5:** SAAT correlation with nerve function and Uni GPA.

Variable	Correlation coefficient	95% CI	*p*-value
Uni GPA	0.272	[0.005, 0.503]	0.0463
b BEL (s)*	0.329*	[0.067, 0.549]	0.015
b BL (s)	0.144	[−0.129, 0.396]	0.298
b BV (m/s)	−0.116	[−0.372, 0.157]	0.401
d BEL (s)	0.205	[−0.066, 0.448]	0.136
d BL (s)	0.229	[−0.041, 0.468]	0.094
d BV (m/s)	−0.166	[−0.415, 0.106]	0.227
a BEL (s)*	0.305*	[0.041, 0.530]	0.025
a BL (s)	0.156	[−0.117, 0.407]	0.259
a BV (m/s)	−0.095	[−0.354, 0.177]	0.492

SAAT, Scholastic Achievement Admission Test (Tahsili); Uni_GPA, university grade point average; B, best reading; b, before pegboard test; d, during pegboard test; a, after pegboard test; (m/s), meter per second; (s), second; E, elbow; W, wrist; V, velocity; L, latency.

*Statistically significant (*p* < 0.05) correlation coefficients.

**Table 6:** Uni GPA correlations with SAAT and age.

Variable	Correlation coefficient	95% CI	p-value
SAAT*	0.272*	[0.005, 0.503]	0.046
Age*	−0.291*	[−0.518, −0.025]	0.032
Average reaction time	−0.085	[−0.345, 0.187]	0.538
BMI	0.003	[−0.265, 0.271]	0.982
GAT	0.164	[−0.108, 0.414]	0.235

Uni_GPA: university grade point average, SAAT, Scholastic Achievement Admission Test (Tahsili); BMI, body mass index; GAT, General Aptitude Test (Qudrat).

*Statistically significant (*p* < 0.05) correlation coefficients.

To determine if body composition influenced the neurophysiological measures, the relationship between BMI and nerve conduction parameters was analyzed. Spearman’s correlation analysis revealed no significant association between BMI and baseline wrist latency (*r* = −0.177, *p* = 0.200) or baseline elbow velocity (*r* = 0.113, *p* = 0.417), suggesting that BMI was not a confounding factor for the observed ulnar nerve conduction variables. A sensitivity analysis restricted to participants with a normal weight (BMI < 25 kg/m^2^, *n* = 30) demonstrated that the direction of the relationship between high school GPA and wrist latency remained negative (*r* = −0.144). However, this association did not reach statistical significance, which is expected given the 45% reduction in sample size and the corresponding loss of statistical power to detect small-to-moderate effect sizes. This further underscores the preliminary, exploratory nature of these secondary findings. Importantly, our previous analysis of the full cohort (*n* = 54) demonstrated that BMI does not significantly correlate with ulnar nerve conduction parameters (*p* > 0.05). Taken together, these results suggest that, while the study is most powered when including the full range of participants, the observed neurophysiological associations are not driven by BMI status. To maintain technical consistency, all ulnar nerve conduction studies were standardized to the non-dominant hand. Prior research by our group has demonstrated no statistically significant differences between the dominant and non-dominant hands regarding ulnar NCV or latency (Almuklass et al., 2024).

## Discussion

The primary objective of this exploratory observational study was to investigate the relationship between ulnar nerve function, as measured by conduction velocity and latency, and measures of academic performance, such as GAT, SAAT, and high school and university GPAs among male medical students. The primary analysis, which examined the association between resting ulnar nerve velocity and university GPA, did not show a statistically significant association. However, secondary association showed a significant inverse correlation between high school GPA and wrist latency. Prior research has shown a similar relationship between nerve conduction velocity and intelligence (Reed et al., 2004). This relationship could demonstrate a more profound association between academic achievement and intellectual processing speed, suggesting that those who are more capable of processing information are more likely to succeed in academic settings. Additionally, we found a significant positive association between SAAT scores and university GPA. These findings align with earlier research showing that early academic achievement is a powerful predictor of future academic performance (Alshammari, 2020). Preparation for the SAAT requires that students build consistent study habits and other skills that may also support university academic performance. Another finding of interest was the negative correlation between age and university GPA, which is consistent with findings from prior research conducted locally (Almalki et al., 2024). Older students may face additional responsibilities such as employment, family, or financial obligations, which limit the time and energy they can dedicate to academic pursuits. These additional obligations can make it difficult for students to be fully involved in their education, resulting in lower academic performance. Unexpectedly, we also found a positive correlation between SAAT scores and elbow latency, both before and after the Pegboard Test. This finding does not support the interpretation that faster nerve conduction is associated with better academic performance. Several explanations are possible, including measurement variability, unmeasured skin temperature, or type I error due to multiple exploratory comparisons. Therefore, this result should be viewed as hypothesis-generating and requires replication before any physiological interpretation. Reaction time was not significantly associated with any academic indicators. Although the simple visual reaction time test is commonly used as a processing speed measure, it captures a small part of the sensorimotor performance and may not reflect the broader cognitive, motivational, and educational factors that contribute to the academic achievement.

Several limitations should be considered when examining the results of the study. First, the study cannot establish a cause-and-effect relationship between peripheral nerve conduction and academic performance due to the study’s observational design. It is not clear whether one influences the other or whether both are affected by other factors. In addition, this study did not directly measure intelligence, cognitive function, or brain activity; GPA, GAT, and SAAT were used only as academic indicators and should be interpreted as indirect proxies rather than direct measures of cognitive ability. Second, the sample was from a single medical college with high performance students with high academic achievement. This suggests a possible ceiling effect and restrict the variability in academic indicators. This may limit the generalizability of the findings. Third, females were not part of the target study population, which resulted in limited representation. Previous research has identified differences between males and females across the domains included in our study. One study reported that females outperformed males on multiple academic variables except on the GAT (El-Moussa et al., 2021). Another study found that ulnar NCV was significantly faster in females compared to males (Nobue and Ishikawa, 2024). Moreover, an earlier study found that females performed better than males in the Grooved Pegboard Test (Almuklass et al., 2016). Recognizing such differences between males and females helps interpret the data precisely and improves the generalizability of the results. In this regard, a similar study that includes only female participants will be conducted to provide a complete picture in both sexes. The exclusion of female participants may have limited the ability to identify sex differences in NCVs that could be significant for a larger group by not involving female participants. Future studies should include female subjects to improve the generalizability of the results. Fourth, the data source was self-reported grades and was not verified against official records, which have major limitations that might affect the validity of study outcomes. The self-reported academic data may have been affected by recall bias because participants might not have remembered the information accurately. Furthermore, social desirability bias can affect self-reported grades, leading individuals to exaggerate their performance to create a favorable image of themselves. This can introduce errors by deliberately overestimating or underestimating actual academic achievement, which may reduce the validity of these data if not verified against official academic records. Fifth, the final sample size was 54, which is smaller than the required minimum sample size of 66. Therefore, the study may have been underpowered to detect small associations. As a result, non-significant findings should be interpreted with caution. Moreover, exploratory correlations were examined without correction for multiple comparisons, increasing the possibility of type I error. Therefore, future studies should involve validated academic data to overcome the limitations of self-reported grades and prespecified statistical models.

## Conclusion

This exploratory observational study found no statistically significant association between the primary variable, ulnar nerve velocity, and university GPA among healthy medical students. However, secondary exploratory analyses showed associations between latency measures and academic performance indicators. The study provides preliminary evidence that peripheral nerve conduction, particularly ulnar nerve latency, may be modestly linked to academic performance in healthy young males. Notably, faster wrist latency during a motor task was significantly associated with higher high school GPA, and standardized test scores such as SAAT correlated with university GPA and some nerve latency measures. Reaction time and nerve conduction velocity showed weaker or no significant associations. Limitations include a small, homogeneous sample, exclusion of participants with higher BMI, and reliance on self-reported academic data, and the findings were not corrected for multiple comparisons; therefore, the results should be interpreted cautiously and require replication. Future research should use larger and more diverse samples, verify academic records, and include brain measures to better understand these relationships. Overall, this study provides preliminary insight into the potential role of neurophysiological measures in academic performance among medical students.

## Data Availability

The raw data supporting the conclusions of this article will be made available by the authors, without undue reservation.
